# ICTV Virus Taxonomy Profile: *Spiraviridae*

**DOI:** 10.1099/jgv.0.001385

**Published:** 2020-01-21

**Authors:** David Prangishvili, Tomohiro Mochizuki, Mart Krupovic, ICTV Report Consortium

**Affiliations:** ^1^​ Institut Pasteur, Archaeal Virology Unit, Department of Microbiology, 75015 Paris, France; ^2^​ Ivane Javakhishvili Tbilisi State University, Tbilisi 0179, Georgia; ^3^​ Earth-Life Science Institute, Tokyo Institute of Technology, Tokyo, 152-8550, Japan

**Keywords:** *Spiraviridae*, ICTV Report, taxonomy

## Abstract

The family *Spiraviridae* includes viruses that replicate in hyperthermophilic archaea from the genus *Aeropyrum *. The non-enveloped, hollow, cylindrical virions are formed from a coiling fibre that consists of two intertwining halves of a single circular nucleoprotein filament. A short appendage protrudes from each end of the cylindrical virion. The genome is circular, positive-sense, single-stranded DNA of 24 893 nucleotides. This is a summary of the International Committee on Taxonomy of Viruses (ICTV) report on the family *Spiraviridae*, which is available at ictv.global/report/spiraviridae.

## Virion

**Fig. 1.**
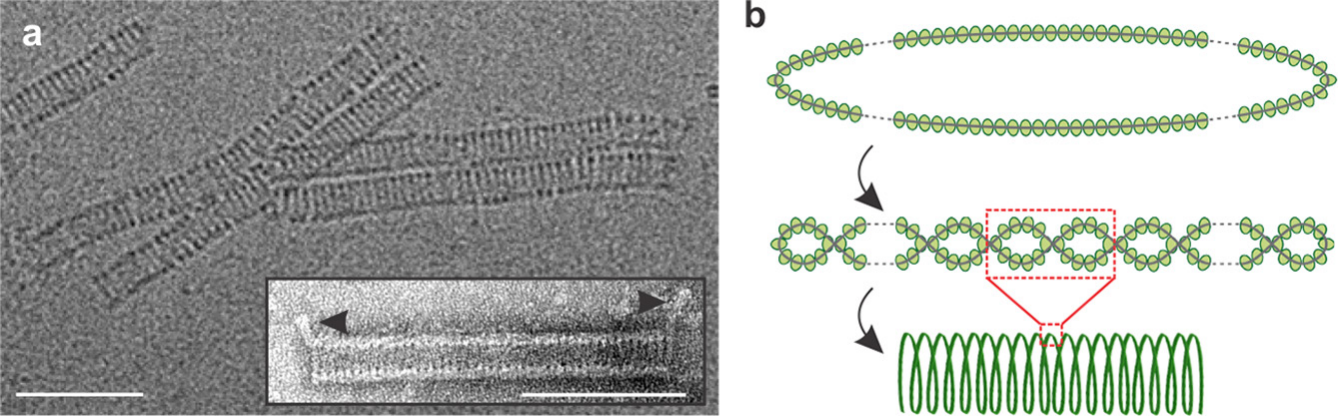
Virions of Aeropyrum coil-shaped virus. (a) Electron micrographs of virions embedded in vitreous ice and (in inset) negatively-stained with uranyl acetate. Arrows indicate appendages at the virion termini. Scale bars, 100 nm. (b) Schematic representation of the different levels of virion organization: the two halves of the circular nucleoprotein (top) intertwine with each other to form a filament (middle), which is condensed into the cylindrical helix of the virion (bottom). Modified with permission from [1].

Virions of Aeropyrum coil-shaped virus are non-enveloped, hollow, cylindrical particles, 230±10×19±1 nm, formed by coiling of a nucleoprotein fibre as a helical spring (Table 1, Fig. 1) [1]. The spring-forming nucleoprotein fibre itself has a helical structure that is formed by two intertwining halves of a circular, single-stranded DNA molecule covered by capsid proteins (Fig. 1). An appendage of 20±2 nm protrudes from each end at a 45° angle to the axis of the cylindrical virion (Fig. 1, inset). The flexible coil of the native virion is prone to contraction and stiffening upon dehydration due to uranyl acetate staining, as is revealed by differences in the size and appearance of virions embedded in vitreous ice and those stained with uranyl acetate (Fig. 1) [1]. Virions have two major proteins with molecular masses of about 23 and 18.5 kDa, and a few minor proteins with molecular masses of 5–13 kDa [1]. The architectural solution used by Aeropyrum coil-shaped virus to package its circular genome is unprecedented among viruses of bacteria and eukaryotes [2]. The coil-shaped morphology represents a group of archaea-specific virion morphotypes [3].

**Table 1.** Characteristics of members of the family *Spiraviridae*

Typical member	Aeropyrum coil-shaped virus (HE681887), species *Aeropyrum coil-shaped virus*, genus *Alphaspiravirus*
Virion	Coil-shaped, 230±10×19±1 nm, with appendages of 20±2 nm at each end; non-enveloped
Genome	Circular, positive-sense, single-stranded DNA of 24 893 nucleotides
Replication	Non-lytic, chronic infection
Translation	Not characterized
Host range	Hyperthermophilic archaea from the genus *Aeropyrum *
Taxonomy	Family *Spiraviridae*, genus *Alphaspiravirus*, single species

## Genome

**Fig. 2.**
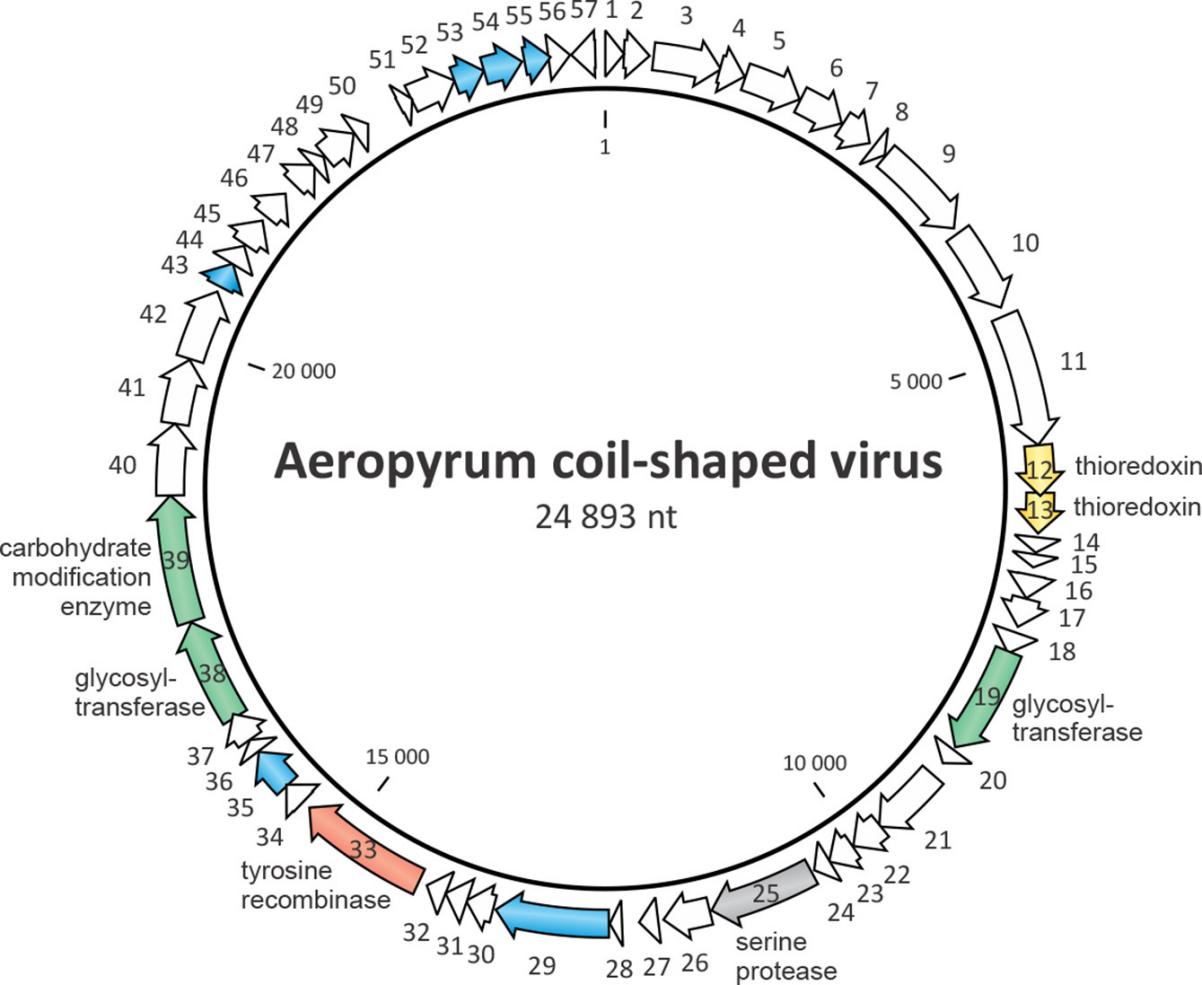
Circular genome map of Aeropyrum coil-shaped virus. The ORFs are marked with arrows indicating the direction of transcription. The ORFs encoding putative proteins for which functions could be predicted are colour-coded and labelled on the figure; blue arrows represent ORFs encoding DNA-binding proteins.

Virions contain a single molecule of circular, positive-sense, single-stranded DNA of 24 893 nucleotides. The G+C content of the genome is 46.7 % [1]. The genome contains 57 predicted open reading frames (ORFs) larger than 40 codons that occupy 93.5 % of the genome (Fig. 2) [1]. The directionality of all but one of these ORFs is the same as that of the DNA strand that is packaged into Aeropyrum coil-shaped virus virions, indicating that the genome is positive-sense. The Aeropyrum coil-shaped virus genome has many more predicted genes than other known ssDNA viruses. Aeropyrum coil-shaped virus encodes a putative trypsin-like serine protease, a tyrosine recombinase, two thioredoxin-like proteins, proteins involved in carbohydrate metabolism and DNA-binding proteins. The virus does not appear to be evolutionarily related to other known archaeal viruses [4, 5].

## Replication

Aeropyrum coil-shaped virus virions are released without apparent host cell lysis. The virus does not encode identifiable DNA and RNA polymerases, nor does it encode any proteins with significant sequence homology with the known Rep proteins involved in the rolling-circle replication mechanism that operates in most of the known single-stranded DNA viruses [6]. Thus, Aeropyrum coil-shaped virus might employ a novel mechanism of genome replication, which is likely to depend on the host replisome.

## Taxonomy

The family *Spiraviridae* comprises a single genus, *Alphaspiravirus*, with one species (Table 1). The virus host *Aeropyrum pernix* is a hyperthermophilic archaeon of the order Desulfurococcales, which grows optimally at 90–95 °C [1, 7]. No relationships with other viruses have been revealed [5]. Aeropyrum coil-shaped virus, along with other archaeal viruses, may represent ancestral virus forms no longer observed amongst extant prokaryotic or eukaryotic viruses [8].

## Resources

Current ICTV Report on the family *﻿Spiraviridae*: www.ictv.global/report/spiraviridae.
